# Microbial Biofuel Cells: Fundamental Principles, Development and Recent Obstacles

**DOI:** 10.3390/bios13020221

**Published:** 2023-02-03

**Authors:** Kasparas Kižys, Antanas Zinovičius, Baltramiejus Jakštys, Ingrida Bružaitė, Evaldas Balčiūnas, Milda Petrulevičienė, Arūnas Ramanavičius, Inga Morkvėnaitė-Vilkončienė

**Affiliations:** 1 Laboratory of Electrochemical Energy Conversion, State Research Institute Centre for Physical Sciences and Technology, Saulėtekio Ave. 3, LT-10257 Vilnius, Lithuania; 2 Faculty of Mechanics, Vilnius Gediminas Technical University, LT-10223 Vilnius, Lithuania; 3 Faculty of Natural Sciences, Vytautas Magnus University, LT-44248 Kaunas, Lithuania; 4 Faculty of Fundamental Sciences, Vilnius Gediminas Technical University, LT-10223 Vilnius, Lithuania; 5 Faculty of Chemistry and Geosciences, Vilnius University, LT-01513 Vilnius, Lithuania

**Keywords:** microbial biofuel cells, yeast, direct electron transfer, extracellular electron transfer, cell membrane/wall modifications, conductive polymers, enzyme-based biofuel cells, bioelectronics

## Abstract

This review focuses on the development of microbial biofuel cells to demonstrate how similar principles apply to the development of bioelectronic devices. The low specificity of microorganism-based amperometric biosensors can be exploited in designing microbial biofuel cells, enabling them to consume a broader range of chemical fuels. Charge transfer efficiency is among the most challenging and critical issues while developing biofuel cells. Nanomaterials and particular redox mediators are exploited to facilitate charge transfer between biomaterials and biofuel cell electrodes. The application of conductive polymers (CPs) can improve the efficiency of biofuel cells while CPs are well-suitable for the immobilization of enzymes, and in some specific circumstances, CPs can facilitate charge transfer. Moreover, biocompatibility is an important issue during the development of implantable biofuel cells. Therefore, biocompatibility-related aspects of conducting polymers with microorganisms are discussed in this review. Ways to modify cell-wall/membrane and to improve charge transfer efficiency and suitability for biofuel cell design are outlined.

## 1. Introduction

**Figure 1:**
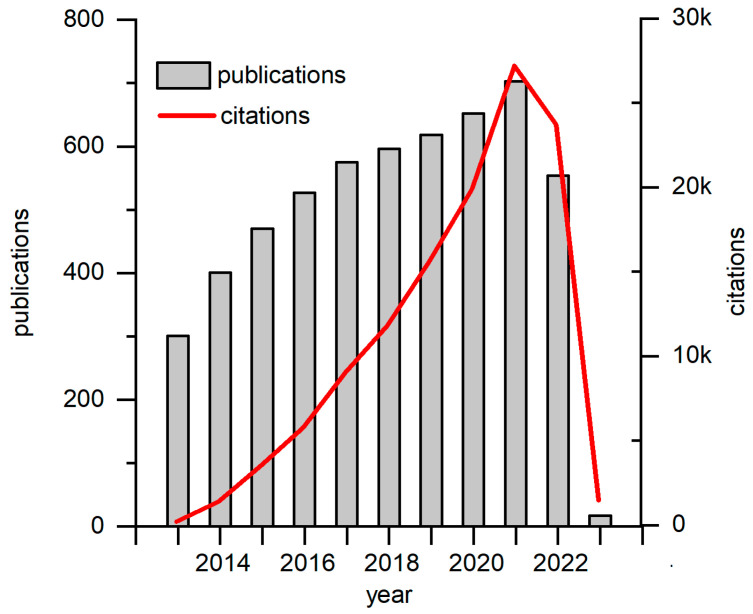
Graphs of recent decade publications and citations on MFC research. Data has been generated on and taken from Web of Science webpage.

Green energy production has recently attracted significant interest from the scientific community. One of the up-and-coming technologies is biofuel cells. Biofuel cells (BFCs) are bioelectrochemical systems or devices that generate electric power by exploiting naturally occurring catalytic or metabolic processes of enzymes, nano enzymes, or even whole cells. Biofuel that can be used can vary from simple high-energy substrates such as glucose, fructose, and saccharose to complex organic molecules. On another hand, BFCs can be applied not only to generate electric power from pure substrates, but also to help treat wastewater by simultaneously producing power and reducing organic waste. Typically, BFCs are classified by their driving force. Enzymatic biofuel cells (EBFCs) [1] use enzymes for energy conversion from substrate stored to electric power. EBFCs show great selectivity towards the substrate and can be implemented for self-powered sensors. However, before use, enzymes need to be purified and efficiently immobilized onto the electrode, thus adding several lengthy, complicated, and expensive steps. Nonenzymatic biofuel cells are a novel idea, where nanomaterials with catalytic properties which mimic natural enzymes are used to produce energy, even though this approach has several advantages compared to EBFCs: high stability, and extended lifetime. However, it suffers from low catalytic efficiency and poor selectivity. Microbial biofuel cells (MFCs) are driven by microorganisms. Such a system allows us to employ the whole metabolic process to be used for energy generation. Hence, multiple substrates or mixed substrates can be used as a fuel source. Additionally, constructed MFCs can renew themselves and prolong the BFCs’ useful life. One of the most novel articles about the use and challenges of using MFCs describes the recent “explosion” in the popularity of these devices [2]. Figure 1 depicts the increasing interest in MFC research.

Furthermore, MFCs compared to other types are less expensive because they can be constructed using microorganisms that are already present in sludge, soil, and other natural habitats. While it has many advantages, the main drawback of MFCs is the inferior capability to transfer charge via cell walls and membranes, which limits widespread adoption. Many efforts have been made to improve MFCs efficiency, primary focus amplifies the charge transfer (CT) from living cells toward the anode. Some of the solutions are the introduction of membrane-bound electron-transferable compounds as intra-/extracellular electron transfer mediators and electrode modifications that improve CT or direct electron transfer from the living cell [1]. The most common microorganisms used in direct electron transfer-based fuel cells are *Shewanella putrefaciens* [3,4], *Geobacter sulfurreducens* [5,6], *Rhodoferax ferrireducens* [7], and *Aeromonas hydrophila* [8]. Metabolic processes and electron transfer mechanisms of these microorganisms are extensively studied, and it is found that physical interaction between the electrode and cytochromes located in the outer membrane or/and conductive layer of bacteria can deliver direct “wiring”. Greater BFCs efficiency is achieved when utilizing microbes capable of producing compounds with redox mediating capabilities rather than using bacteria incapable of producing such compounds [1,9].

Eucaryotic cells used for MFCs are not yet common. However, eukaryotic microorganisms such as *Saccharomyces cerevisiae* are highly investigated for use as MFCs biocatalysts [10,11,12]. *S. cerevisiae* has many desirable characteristics such as a broad substrate range, well-known metabolic pathways, simple and rapid mass cultivation, and affordable prices, which simplifies MFCs construction. Even so, *S. cerevisiae* naturally does not produce compounds with redox mediating capabilities, so the system requires the addition of redox mediators to perform efficiently [11,12,13,14,15]. The redox mediator, a molecule positioned within the cell membrane, is easily accessible to NADH and can join the anaerobic glycolysis NADH/NAD+ redox cycle [10]. The addition of a redox mediator does not hinder the normal metabolism of the cell and energy can be extracted when NADH is re-oxidized into NAD+ while a redox mediator gets reduced. Additionally, to enhance *S. cerevisiae*-based MFCs, different modifications of electrodes, cell walls, and membranes can be applied [1,16,17,18].

This paper overviews some recent developments in the design of microbial biofuel cells.

## 2. MFC Working Principles

**Figure 2:**
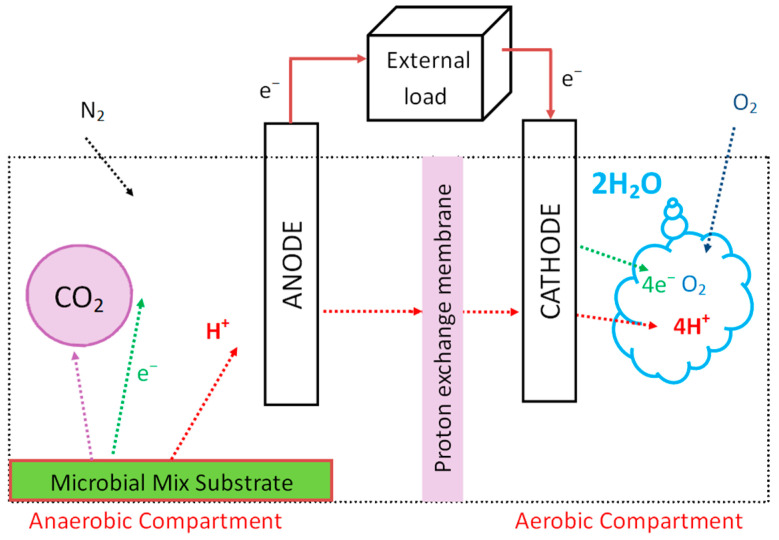
Scheme of the microbial fuel cell. The electrodes are connected by a wire and electrons from the redox reactions in the anode compartment are passed through the wire to the cathode. As the microbial mix substrate is providing protons in the anaerobic chamber to transfer to the aerobic one, the electrical circuit is complete, and the charge is generated.

MFCs have two compartments: (1) anode and (2) cathode (Figure 2). Compartments are separated by a membrane through which protons are transferred from the anode to the cathode compartment. Charges (protons and electrons) in the anode compartment are released from the metabolic activity of microorganisms during the microbial oxidation reaction of a substrate that is the fuel of MFCs. Electrons are transported via the anode to the cathode through an external load. At the cathode, an oxygen reduction process takes place in which electrons react with protons and oxygen to form water. MFCs typically use glucose as a fuel, which has a high energetic value and can generate 0.3–0.5 volts [19].

Currently, there are limited resources on MFCs applications since they are in their early development stages. The most important issue is low power density output coming from inefficient CT. The most popular anodes from carbon felt provide a large surface area, which can be modified with electrically conductive materials to improve CT. Moreover, they have good surface physical characteristics suitable for microbial attachment which can result in more efficient direct electron transfer from biocatalyst to anode [20,21]. The highest power density was achieved by using carbon felt anode decoration with gold nanoparticles [21], manganese oxide, or iron oxide nano-flowers [20], following modification by *S. cerevisiae* (Table 1). In both cases, *S. cerevisiae* combined with an electrode surface covered with nanostructures provided a durable direct electrochemical ‘wiring’. In many cases, *S. cerevisiae* was used as a model system. Nevertheless, the microorganism can be chosen depending on the available fuel, or the purpose of the whole system.

For example, an organic toxin para-aminophenol is an excellent fuel for the *S. dehoogii*-based MFCs. Such MFC could be used to reduce this toxicity in wastewater, and it can operate for up to eight days [22,23]. The MFCs based on *S. loihica* could be used as a nontoxic and environmentally friendly method for the remediation of chromium, and its pollution by the production of chromium nanoparticles since *S. loihica* is known to reduce metals [24]. Furthermore, MFCs based on *Negativicutes* and *Gammaproteobacteria* were used for purification and energy generation from sewage silt [25]. Many papers emphasize the modification of electrode surfaces with different materials for improved performance [14,18,20,21,26,27,28].

## 3. Mediators Used in MFCs

MFCs nowadays produce relatively low current and power, even though significant amounts of energy could be created during the metabolic redox process that takes place in live microorganisms while purifying waste [24,29,30,31,32]. The main limiting factor is hindered CT capacity by the cell walls and membrane [32]. It can be significantly improved using appropriate redox mediators [32]. Redox mediators can be hydrophilic or lipophilic, [33,34], and redox polymer-based matrices [32,35,36]. Some of the best-performing artificial mediators (including thionin, methylene blue, and neutral red) have been reviewed in [37].

Hydrophilic mediators usually enhance MFC performance by interacting with the cell trans-plasma redox system [38]. This interaction takes place between cytoplasmic mediators and redox enzymes. The most typical examples are membrane-bound cytochromes [39]. Cytochromes typically carry a co-enzyme, a functional group, a redox-active center, or a combination of them [40]. Generally, the hydrophilic mediator cannot pass through the membrane. Hence, there is a need for lipophilic mediators that play the vital role of CT through the cell membrane. These redox mediators can dissolve into the plasma membrane and can easily transport charge from cell internals to the outer leaflet of the cell membrane. Lipophilic mediators execute CT via functional groups. When lipophilic mediators in combination with hydrophilic are used, a significant improvement in CT is achieved [38]. As lipophilic mediators, several quinones can be used. Their drawbacks, however, include toxicity and damage to microbial cells which can vary significantly depending on their chemical properties and the conditions of cellular exposure [41,42,43,44,45]. For example, 9,10-phenanthrenequinone (PQ), which is a lipophilic redox compound, can be used in MFC, while PQ is immobilized on the anode, and hydrophilic ferricyanide can be dissolved in a working solution [11,12,46].

Metal or carbon-based nanomaterials can be synthesized in the required size and shape to facilitate CT from cells’ metabolic processes [14,47,48]. Nanoparticles form an electric channel from the cell to the electrodes. Modification with nanoparticles can take place either on the electrodes or by cell conjugation with nanoparticles [49,50]. In such systems, it is crucial that the nanocomposites do not kill the microorganisms [51]. Hence, only biocompatible nanomaterials should be used. These substances, such as gold nanoparticles [52] or carbon nanotubes [53], enhance the CT of the final MFC product. Mediators used in MFCs should have the following properties:

1. Electrochemical activity.
2. Biocompatibility with microorganisms used in the MFCs.
3. Cell membrane permeability.
4. Redox potential should be suitable for mediated electron transfer.
5. Stable and soluble in both oxidized and reduced forms.
6. Fast oxidation kinetics at the electrode surface [32,37].

Our investigations of MFCs mostly include yeast. Hence, Table 1 represents the most often utilized mediators for this kind of cell culture. Based on the data, Methylene Blue and Tetramethyl-phenylenediamine seem to transfer the most power output, 500 and 1000 mW m^−2^ maximum accordingly, although others are also considerable for utilization and the transferred power output varies from 0.408 to mentioned 1000 mW m^−2^.

## 4. Modification of Microorganisms by Conductive Polymers

**Figure 3:**
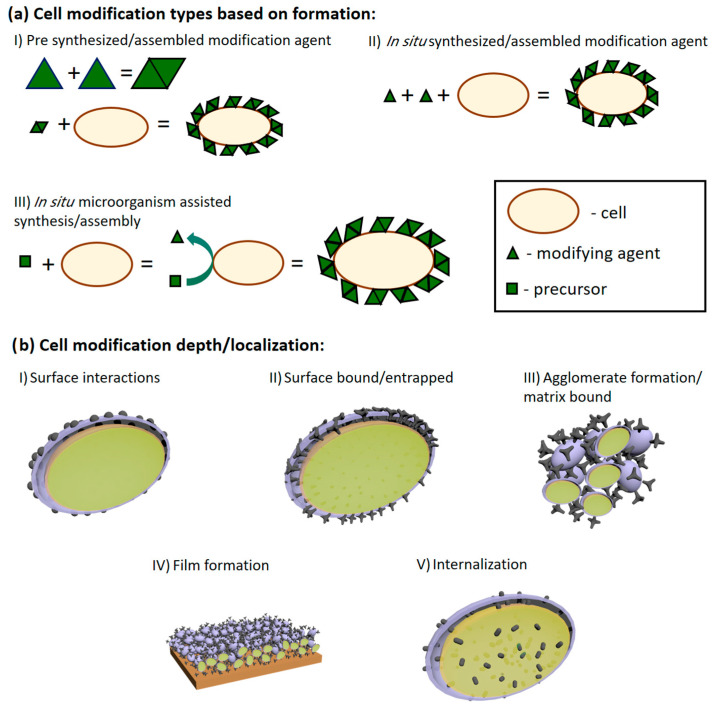
(**a**) Schematic depiction of cell modification by agent formation principle (**a**). Cells can be modified using pre-synthesized compounds (I), assembled/synthesized in situ in the presence of living cells (II), and in situ when the cells assist/catalyze the synthesis assembly of the modifying agent (III). (**b**) Schematic representation of modifying agent localization in MBFC applications: (I) surface interactions as adsorption and electrostatic interactions; (II) modifying agent is either covalently bonded or forms interlacing and inseparable structures with cell walls or other similar structures; (III) when modifying agent forms aggregates from its matrix and cells; (IV) higher agglomerate organization onto surfaces; (V) internalization of modification agent. In picture (**b**), the purple surface represents the cell wall; the light green part of the picture represents the inside of the cells; the dark green parts of the picture show the modifying agents. Adapted from [7].

Electrochemical sedimentation of conductive polymers (CPs) is a relatively simple method for modifying electrode surfaces and has become a popular choice when designing bioelectronic devices [67] (Figure 3). Its popularity comes from the ease of controlling physical characteristics, where layer thickness, density, and ion permeability can be adjusted by changing the electrochemical conditions required for the polymerization reaction [15,68,69]. Biologically active molecules, such as proteins [70,71,72,73], DNA [74,75], and even live cells and bacteria, can be immobilized within CP layers. However, it is important to mention that other chemical factors: solvents, monomers, polymerization bulk composition, and pH, also have a significant impact on the features of produced CP layers.

**Figure 4:**
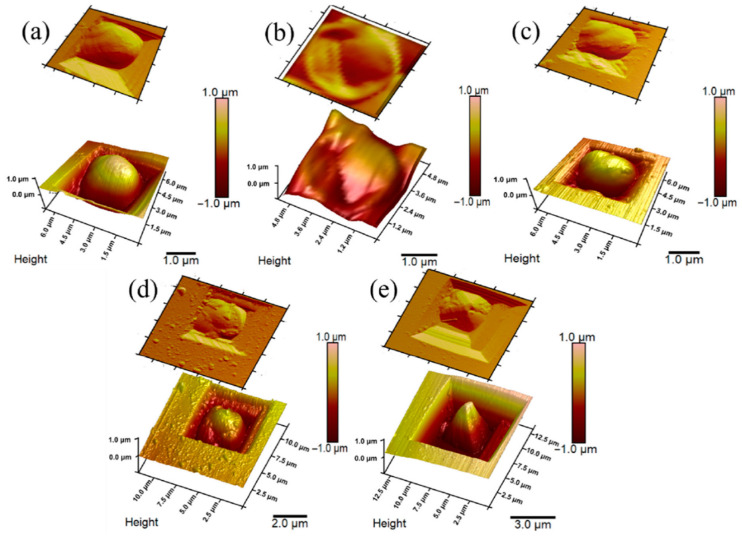
AFM images: (**a**) non-modified yeast cell; (**b**) inactivated yeast cell; (**c**) PPy0.05-modified yeast cell; (**d**) PPy0.1-modified yeast cell; (**e**) PPy0.3-modified yeast cell. Adapted from [46].

Conductivity is one of the most important characteristics when designing highly efficient bioelectronic devices. Some research groups established methods to evaluate the conductivity of electrochemically deposited polypyrrole (PPy) layers [76] and polyaniline (PANI)-based layers [75,77]. Our group proposed a mathematical model [78] to predict the conductivity of formed layers. Using this information, properties of multi-PPy layer electrodes can be predicted. Therefore, only the most efficient structures can be constructed. Even though CP layers may increase efficiency [79,80,81], formed layers on the electrode itself could hinder the diffusion of nutrients and destabilize metabolic processes. To relieve this problem, organic ‘spacers’ are introduced to CP layers to alter the porosity while interlinking various polymeric chains [82]. For this reason, several types of microorganisms were entangled in the structure of various polymers, including conductive polymers [11,12,17,46,75,83,84] (Figure 4).

**Figure 5:**
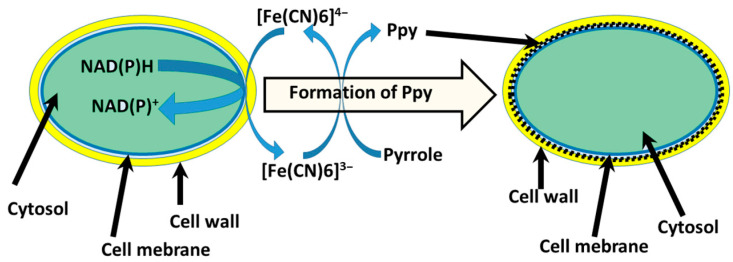
Schematic representation of PPy synthesis in the cell wall of yeast [13]. Redox enzymes located in the plasma membrane oxidize [Fe(CN)_6_]^4−^ into [Fe(CN)_6_]^3−^ and induce a polymerization reaction of pyrrole [95].

However, the electron transfer from microorganisms to the electrode is infrequently observed even when various microorganisms [85,86] and mammalian cells (specifically lymphocytes [87] and erythrocytes [88]), are used for BFCs construction. To enhance the electron transfer, microorganisms could be modified with CP (Figure 5). Cells can be modified by exploiting their metabolic processes to initialize the polymerization of CPs [13,14,84,89]. Microorganisms (yeast, stem cells) modified with CPs typically preserve their viability, remain metabolically active, and form a stable system [90,91]. It was found that such systems’ functional lifetime is prolonged compared to polymer-modified enzymes [92,93,94].

Currently, the PPy application is gaining extra attention in the field of cell self-encapsulation [89]. Polymer matrices can be prepared in situ with cell culture or produced through metabolic/chemical processes within the cell structure. To our best knowledge, the first work on PPy bio-assisted polymer synthesis was performed in 2016 by our group [83]. The capacity of *Streptomyces* spp. to release redox enzymes (e.g., phenol-oxidase) to extracellular media enables the bacteria to initialize the creation of spherical PPy particles without the need for additional chemicals. For instance, it was identified that phenol-oxidases could be used to synthesize polypyrrole. After six days of multiplying, *Streptomyces* spp. bacteria favorable conditions are established for the arrangement of hollow PPy microspheres with a diameter of 10–20 µm [83]. Particle shapes appeared to have been influenced by organic compounds present in the growth medium [83].

Afterward, it was described that encapsulation of yeast *S. cerevisiae* cells by PPy could be achieved [13,95]. In this instance, yeast cell metabolic processes were employed to cycle redox mediator ([Fe(CN)_6_]^4−^/[Fe(CN)_6_]^3−^) that initializes the polymerization process in situ under controlled conditions (Figure 5). Cell shape, diameter, and roughness of the surface after the modification with PPy are related to the viability of cells [46]. Designing MFC based on CP-modified microorganisms’ cells that sustain viability after the modifications is the most desirable. Increasing the concentration of pyrrole during the modification stage causes cells to become smaller in diameter, surface roughness also increased, and small clusters of formed polymers can be observed. A minimal change in cells’ physiological state was observed at the lowest 0.05 M pyrrole concentration, suggesting yeast cells sustained their viability. Therefore, the system was used for the MFC design. Constructed MFCs generated power (47.12 mW/m^2^), compared to the non-modified system, was higher by 8.32 mW/m^2^.

In addition to yeast modification, methodologies to achieve similar results were developed. By introducing iron nitride, iron (III) nitrate nonahydrate, various bacteria: *Streptococcus thermophiles*, *Ochrobacterium anthropic*, *Escherichia coli*, or *Shewanella oneidensis*, MR-1 can form similar PPy layers [96]. In preparation, bacterial cells were saturated with iron (III) nitrate nonahydrate, which was placed in cell outer layers, and then polymerization was initiated upon the addition of pyrrole [96]. It was reported that bacterial cells retained viability, and the coating procedure did not affect cell proliferation. Moreover, in terms of electrical characteristics, the treatment of cells with conductive polymer (PPy) has resulted in a 14,1-fold improvement in power density comparison to unmodified *S. oneidensis* (147.9 µW cm^−2^) [96]. Analogous self-encapsulation was applied for microorganisms *Aspergillus Niger* and *Rhizoctonia* sp., and successfully used for MFC applications [32,84,97].

Scanning electrochemical microscopy (SECM) could be applied for MFC assessment [89]. During electrochemical probing over immobilized modified white-rot fungal cell culture, the current production (I_max_ = 0.86 nA) was nearly three times higher than control groups (I_max_ = 0.30 nA) [89]. Results were obtained from the surface approach curves. In addition, these studies revealed that the charge transfer efficiency, which is critical for the current production of MFC, is dependent on several variables: (1) the distance between the ultra-micro electrode of SECM and the cells and (2) the modification of microorganisms. The current recorded when the ultra-micro electrode distance from the sample surface was 20 µm (0.47 nA) was 1.5 times greater compared to the control sample [97]. Researchers noted that PPy production in fungal hyphae was facilitated by the laccase enzyme, which *Trametes* spp. fungus synthesizes and releases into the growing media. Utilizing crude enzyme extract with cell culture in a nutrient broth, polymerization of pyrrole was detected. At that time, bio-assisted polymer synthesis was very novel [98], and to the best of our knowledge, this was one of the first research that enabled the practical use of enzyme-assisted creation of conductive polymers [98,99,100,101,102]. Later, it led to polymer-based coating formation in cell culture [13,14,84,89]. Thus, it was demonstrated that cells modified with conductive polymer have advanced electron transferability, which enables to use of these microorganisms in microbial biofuel cells (MFCs) [32].

Furthermore, researchers reported that bacteria capable of metal reduction: *Clostridium sporogenes*, *Cupriavidus metallidurans,* and *Escherichia coli*, could use FeCl_3_ to initialize atom transfer radical polymerization of (poly(ethylene glycol methyl ether methacrylate); N-Hydroxyethyl acrylamide; hydroxyethyl methacrylate; 2-(methacryloyloxy) ethyl dimethyl-(3-sulfopropyl) ammonium hydroxide and 2-Acrylamido-2-methyl-1-propane sulfonic sodium) [103]. These cultures reduce Fe^+3^ to Fe^+2^ in a controlled way and initiate the polymerization of monomers. It is important to mention that monomers must be nontoxic to cells and engage in redox processes of Fe^+2^/Fe^+3^. After polymerization cells preserve high viability [103]. Along with PPy, various polymers are also employed to improve the performance of MFCs. Similarly, *S. xiamenensis* were coated with polydopamine (PDA) [103]. Selected bacteria can adhere to PDA during biofilm on MFC formation via oxidative polymerization in aerobic and slight alkali (pH 8) conditions. Researchers reported that PDA-modified bacteria *S. xiamenensis* cells were able to generate a much higher 452.8 mW/m^2^ power density, which was 6.1 times greater than the MFC system using nonmodified cells (74.7 mW/m^2^) [103]. Moreover, within three hours, conductive PDA additives were generated, which is quite quick. In addition, it appears that the modification of bacteria had insignificant effect on cell viability, which decreased only by 2–3% [103]. A prevalent bacterium for MFC design is *Shewanella oneidensis* MR-1, coated with PDA. In their study [104], Yu et al. reported that it is possible to use cell-assisted synthesis to form conductive PDA and use the same bacteria to exploit the biomineralization of FeS nanoparticles. Results showed that different interfaces wire up a cell at different levels. Thus, their electric/electrochemical properties are different. Polysulfide reductase mineralized FeS nanoparticle interface boosted the efficiency of MFC anodes up to 3.2 W/m^2^, and this was 14.5 times more than anodes modified by native *S. oneidensis* cells (0.2 W/m^2^), although the power output of PDA coated anodes was roughly 0.6 W/m^2^ [104].

Researchers developed an alternate strategy by internalizing the feeding process of pre-synthesized carbon dots (CD) and carbon nanoparticles into *S. oneidensis* and *Shewanella xiamensis*, respectively [105,106]. Both studies showed remarkable effects of CD that turned out to be highly biocompatible. Furthermore, CD could enhance metabolic activity by significantly increasing internal ATP (Adenosine 5′-triphosphate) levels. Overall, it was believed that a boosted metabolic rate might generate harmful reactive oxygen species. However, it was not the case. Moreover, CD generated photoactive particles that stimulate lactate consumption and result in a current generation when illuminated. With Shewanella oneidensis, MR-1’s maximum current density of 1.23 A/m^2^ was achieved, compared to the control of 0.19 A/m^2^ [105]. Meanwhile, the maximum power density of the MFC with CD was 0.491 W/m^2^ and was 6.46 folds higher compared to the control using the same nonmodified bacteria (0.076 W/m^2^). Shewanella xiamenensis attained a current density of 329.4 µA/cm^2^ under illuminance with lactate (as the only carbon source), which was 4.8-fold more than the control (68.1 µA/cm^2^) [106].

Osmium redox polymers can also be applied in developing MFCs [27,107,108,109,110]. The researchers attained the highest charge density of 15.079 mA/cm^2^ and an open circuit potential of 176 mV.

To compare the employed anodes and their materials for MFCs, as well as the power density of each version, the data in Table 2 are presented, with the order from lowest to highest power density.

Here, we explored and overviewed emerging technologies and methodologies for enhanced performance of MFCs by introducing some agents into cells themselves or covering them. In summary, these technologies fall under the headings of cell surface engineering, internalization, and artificial biofilm synthesis. Polymeric coating formation and polymer inclusion inside live cells represent the most promising approaches for cell manipulation. Even though there is clear proof of such a modification-based influence on charge transfer [32], there are still a few disadvantages. Some improvements are rather intricate, and their implementation in real-world MFCs might be challenging [27,107,108,109,110]. The primary disadvantages are microbe survival and proliferation since newly created cells in MFC must either inherit the change or experience it. These drawbacks compromise the longevity and stable electricity output of the MFCs. Consequently, the net electricity output of MFCs should be increased by a synergistic impact resulting from the combination of cell surface changes and other techniques described in this paper.

## 5. Conclusions and Future Aspects

Microbial fuel cells (MFCs) are a developing technology suitable to produce ‘green’ power and support bioremediation for the rising use of fossil fuels generating a worldwide energy crisis and a heightened awareness of environmental issues. However, the power generated by MFCs is still low for practical applications. Thus, MFC performance must be enhanced. The anode and current-generating bacteria are two crucial MFC structural components. The anode arranges the medium for microorganism attachment, while the living cells undertake bacteria-electrode charge transfer mechanisms. The low performance of the anode in MFC is the most significant challenge for its proper utilization these days. Effective anode modifications are presumed to increase the surface area and provide for the efficient attachment of biofilm, which subsequently intensifies the electrical power production by MFC. The microorganism-based biofuel cells show a relatively poor power density, mainly because charge transfer from the cells to the electrodes is restricted. These restrictions are caused by natural cell barriers (membrane and cell wall) that insulate the cell from an outer environment. This inconvenience can be very effectively exploited in the structure of microbial biofuel cells, as the immobilized cells can use various materials for fuel to generate electrical energy and be compatible with high cell viability and metabolic activity. To significantly increase the electron transfer rate and power density of MFCs, several chemical modifications of the cell wall or membrane are used. Carbon nanotubes, conductive polymers, metal nanoparticles, and other metal-based nanostructures have been used to increase the performance of MFCs by modifying the anode and different cell walls and plasma membranes.

Electrochemically covering the electrodes of BFCs with conductive polymers, such as PPy or PANI, or the mixtures of conductive polymers with chitosan or hydrogels might alleviate the biocompatibility difficulties of implantable MFCs. Furthermore, diverse properties of produced layers may be readily manipulated by selecting appropriate chemical and electrochemical conditions for effective electrode modification to minimize inflammatory responses while in touch with human tissues.

In summary, the future of BFCs and MFCs seems promising since the capabilities of such devices’ applications are immeasurable. Furthermore, although designs of BFCs and MFCs deliver poor electrical output, various studies have shown that there are no limits to their diversity. Each modification suggests a novel approach to the higher efficacy of these devices, and each modification leads one step closer to the application of such devices in daily life.

## Figures and Tables

**Table 1:** Description of the performance of various yeast-based MFCs using respective mediators for the systems. Abbreviation YEPD stands for “yeast extract peptone dextrose”.

Yeast	Substrate	Mediator	Anode	Power Output (mW m^−2^)	Ref.
*S. cerevisiae*	Glucose	Resorufin	Glassy carbon	155	[54]
*S. cerevisiae*	Glucose	Methylene blue + K3[Fe(CN)6]	Reticulated vitreous carbon	147	[55]
*S. cerevisiae*	Glucose	Thionine	Graphite	60	[56]
*S. cerevisiae*	Glucose	Neutral red	Graphite plate	133	[57]
*S. cerevisiae*	Glucose	Methylene blue	Platinum mesh	65	[58]
*S. cerevisiae*	Glucose	Methylene blue	Copper electrode	4.48	[59]
*S. cerevisiae*	Dextrose	Methylene blue	Reticulated vitreous carbon	400	[60]
*S. cerevisiae*	Dextrose	Neutral red	Reticulated vitreous carbon	100	[60]
*S. cerevisiae*	Dextrose	Methylene blue with Neutral red	Reticulated vitreous carbon	500	[60]
*S. cerevisiae*	Dextrose	Methylene blue	Carbon felt	300	[61]
*S. cerevisiae*	YEPD with glucose	Methylene blue	Carbon felt modified with poly-ethyleneimine	429.29 ± 42.75	[62]
*S. cerevisiae*	YEPD with glucose	Methylene red	Carbon felt modified with poly-ethyleneimine	282.77 ± 15.95	[62]
*S. cerevisiae*	Glucose	Menadione + K3[Fe(CN)6]	Graphite rod	0.408	[11]
*S. cerevisiae*	Glucose	9,10-phenantrenequinone + K3[Fe(CN)6]	Graphite rod	22.2	[12]
*C. melibiosica*	YEPD with fructose	Bromocresol green	Carbon felt	46	[37]
*C. melibiosica*	YEPD with fructose	Methyl orange	Carbon felt	137	[37]
*C. melibiosica*	YEPD with fructose	Methyl red	Carbon felt	113	[37]
*C. melibiosica*	YEPD with fructose	Neutral red	Carbon felt	89	[37]
*C. melibiosica*	YEPD with fructose	Methylene blue	Carbon felt	640	[37]
*C. melibiosica*	Fructose	Methylene blue	Graphite rods	185	[63]
*C. slooffiae strain JSUX1*	Xylose	Riboflavin	Carbon felt	67	[64]
*P. fermentans*	YEPD broth	Methylene blue	Carbon fibers in dual chamber	12.3	[65]
*P. fermentans*	YEPD broth	Methylene blue	Carbon fibers in single membrane-less chamber	16.4	[65]
*A. adeninivorans*	Dextrose with glucose	Tetramethyl-phenylenediamine	Carbon fibre cloth	1000	[66]

**Table 2:** Description of the MFC anode modification method and performance. Abbreviations are provided below the table [7].

Anode	Anode Material/Electron Donor	Power Density, mW m^−2^	Ref.
*Bacillus subtilis* on aldrithiol monolayer and OsRP	Gold, Graphite/Succinate	-	[27]
*Saccharomyces cerevisiae* on PQ and MWCNTs	Graphite/Glucose	1.13	[14]
*Saccharomyces cerevisiae*	Carbon paper/Glucose	3	[111]
*Scedosporium dehoogii*	CF/APAP	6.5	[22]
*Shewanella loihica* on PANI and carbon nanotubes	APTES, ITO/Sodium lactate	34.5	[112]
*Scedosporium dehoogii*	CF/APAP, Lignin	50, 16	[23]
*Saccharomyces cerevisiae* with CNTs	PU/Glucose, MB	100	[18]
*Thermincola ferriacetica*	Graphite/DSMZ	146	[113]
*Saccharomyces cerevisiae* on PEI and one of the QS molecules (phenylethanol, ryptophol, and tyrosol).	CF/Glucose	159 * 156 135	[28]
*Gammaproteobacteria* and *Negativicutes* on MWCNTs blended with biogenic Au	CF/Sludge	178	[25]
*Saccharomyces cerevisiae* on PEI and CNTs	CNTs/Glucose	344	[26]
*Escherichia coli*	Platinized titanium/Glucose	502	[114]
*Candida* *melibiosica*	CF/Methylene blue	640	[37]
*Pseudomonas aeruginosa*	Chitosan, vacuum-stripped graphene/Glucose	1530	[115]
*Saccharomyces cerevisiae* on PEI and AuNPs	CF/Glucose	2771	[21]
*Saccharomyces cerevisiae* on alginate	CF/Glucose	3900	[116]
*Saccharomyces cerevisiae* on PEI, with SDBS and FeMnNPs	CF/Glucose	5838	[20]

*—power densities for MFCs based on phenylethanol, ryptophol, and tyrosol, respectively; APAP—acetaminophen; APTES—γ-aminopropyltriethoxysilane; CF—carbon felt; DSMZ—Deutsche Sammlung von Mikroorganismen und Zellkulturen (bacteria growth medium); MB—methylene blue; MWCNTs—multi-walled carbon nanotubes; NPs—nanoparticles; OsRP—osmium redox polymer; PEI—polyethylenimine; PU—polyurethane; PQ—9,10-phenanthrenequinone; QS—quorum sensing.

## Data Availability

Not applicable.
